# Friedelin Ameliorates Nucleus Pulposus Inflammation by Increasing p65 Autophagic Degradation to Inhibit NF‐κB Signalling Pathway

**DOI:** 10.1111/jcmm.70989

**Published:** 2026-01-08

**Authors:** Kewu Tu, Dongteng Liao, Zhaomou Chen, Zhenyu Wang, Kun Zhao, Hongyu Zhong, Shimin Wu, Huiyin Zhu, Jinming Xu, Beidi Zhou, Xiangheng Dai, Qiang Wu

**Affiliations:** ^1^ Department of Spinal Surgery, Shaoguan First People's Hospital Guangdong Medical University Shaoguan China; ^2^ The First School of Clinical Medicine Guangdong Medical University Zhanjiang China; ^3^ Department of Orthopaedics Guangzhou Red Cross Hospital of Jinan University Guangzhou China; ^4^ Second Clinical School Guangzhou Medical University Guangzhou China; ^5^ Medical School of Shaoguan University Shaoguan China; ^6^ Department of Joint, Sports Medicine & Pediatric Orthopedics, Shaoguan First People's Hospital Guangdong Medical University Shaoguan China; ^7^ Department of Prevention and Health Care, Shaoguan First People's Hospital Guangdong Medical University Shaoguan China

**Keywords:** Friedelin, inflammation, intervertebral disc degeneration, NF‐κB signalling, RNF182

## Abstract

Intervertebral disc degeneration (IVDD) is a prevalent disorder associated with chronic inflammation, significantly affecting spinal health and general quality of life. This study examines the anti‐inflammatory properties of Friedelin (FD) and its impact on the NF‐κB signalling pathway in relation to IVDD. In vivo experiments utilising cervical intervertebral disc tissue from mice exhibiting cervical spine instability and in vitro assays with nucleus pulposus (NP) cells revealed that FD treatment significantly diminished NP degeneration and inflammatory cytokine production, concurrently inhibiting NF‐κB activation. FD triggered autophagic clearance of p65, reducing inflammatory cytokine output. This effect was mediated by selective inhibition of p65 phosphorylation, independent of IKK activity, highlighting its targeted action on NF‐κB signalling. Moreover, FD enhanced the association between p65 and the E3 ubiquitin ligase RNF182, facilitating p65 degradation via autophagy. The findings indicate that FD mitigates IVDD by diminishing NP degradation and inflammation while also presenting a potential therapeutic strategy that targets the NF‐κB signalling pathway through autophagic processes.

## 1 Introduction

Intervertebral disc degeneration (IVDD) is a common ailment that considerably affects spinal health and overall quality of life [1]. This condition entails the degeneration of intervertebral discs, potentially leading to persistent pain, incapacity and reduced mobility [2]. Recent studies underscore that chronic NP inflammation drives IVDD progression [3, 4, 5]. Despite this understanding, the specific molecular mechanisms underlying the inflammatory response in IVDD remain poorly defined and effective therapeutic options are limited [6, 7]. This highlights a significant gap in current knowledge and underscores the urgent need for further research to develop better treatments.

Recent investigations indicate that intervertebral discs undergo several biochemical alterations during degeneration, including changes in the extracellular matrix and heightened production of inflammatory cytokines [8, 9]. The nuclear factor kappa B (NF‐κB) signalling pathway is a vital component in this process, recognised as a fundamental regulator of inflammation in IVDD [10, 11]. Many mediators and pro‐inflammatory cytokines that aid in degeneration are controlled by this route [12, 13]. Thus, targeting the NF‐κB pathway may be a viable therapeutic approach to mitigate the inflammatory response and possibly decelerate the evolution of IVDD.

Friedelin (FD), a triterpenoid compound, has garnered attention for its potential anti‐inflammatory properties [14, 15, 16]. Prior studies have demonstrated that FD can safeguard against inflammation and degeneration in several tissues [15, 17]. Preliminary research into the mechanisms of FD indicates that it may influence the NF‐κB pathway, hence warranting its exploration as a potential treatment agent for IVDD. The exact molecular pathways by which FD mediates its anti‐inflammatory benefits in IVDD remain inadequately elucidated, highlighting a crucial domain for further investigation.

This work used a combination of in vivo and in vitro methodologies to investigate the impact of FD on inflammation and degeneration in the nucleus pulposus (NP). We examine cervical intervertebral disc tissue samples from mice exhibiting cervical spine instability (CSI) and cultured NP cells to elucidate the impact of FD on inflammatory cytokine production, NF‐κB activation and the associated molecular pathways. This thorough method offers enhanced understanding of the impact of FD on IVDD and its possible therapeutic uses.

The main aims of this research are to evaluate the efficacy of FD as an anti‐inflammatory drug in IVDD and to elucidate its potential impact on the NF‐κB signalling system. By investigating the relationship between FD treatment, NP degradation and the synthesis of inflammatory cytokines, we seek to elucidate how FD may mitigate the effects of chronic inflammation linked to IVDD. This study seeks to further the creation of innovative therapeutic approaches for the treatment of IVDD and enhance patient outcomes.

The increasing prevalence of IVDD underscores the pressing necessity for a deeper comprehension of its fundamental mechanisms and the exploration of innovative therapy strategies. This research seeks to fill critical knowledge gaps by focusing on the anti‐inflammatory properties of FD and its interactions with the NF‐κB signalling system, hence enhancing prospective therapy alternatives for IVDD.

## 2 Materials and Methods

### 2.1 Materials

Friedelin obtained from MedChemExpresswas (Shanghai, China; HY‐N4110). Additional significant reagents and antibodies are enumerated in Tables S1 and S2.

### 2.2 Animals, IVDD Model and Treatment

Animal experiments were approved by the Southern Medical University Animal Care and Use Committee (SMUL2021014). Forty male C57/BL6 mice, each 10 weeks old and approximately 25 g, were acquired from the Experimental Animal Centre of Southern Medical University. CSI surgery was performed under isoflurane anaesthesia, following previously described procedures [18, 19]. Mice were initiated in adult rats with 4% isoflurane in 100% O_2_ and maintained at 2% isoflurane delivered through a Matrix VIPl00 small‐animal anaesthesia system (Matrix, USA). The anaesthetised animal was positioned prone and the skull secured in a stereotaxic frame. After shaving and disinfecting the dorsal cervical skin, the C2 and T2 spinous processes were identified as external landmarks. A 3‐cm midline incision was made between these landmarks. Fascia and paraspinal muscles were bluntly dissected along their natural cleavage planes and retracted with a mini‐retractor to expose the laminae from C3 to T1. Remaining soft tissue on the laminae was carefully removed with a micro‐blade, preserving the medial aspects of the facet joints. In the laminectomy groups, bilateral laminectomies from C3 to T1 were performed with a fine double‐action rongeur, avoiding cord compression or dural trauma. Haemostasis was achieved with gentle pressure and, when necessary, micro‐gel foam. Layers of muscle and fascia were closed, and 4‐0 nylon was used to stitch the skin. Sham‐operated mice underwent the same incision and muscle dissection without laminectomy. All mice (*n* = 40) were supplied intramuscular penicillin (400,000 U/Kg) daily for 3 days post‐surgery and meloxicam (5 mg/kg) was given subcutaneously immediately after the procedure to manage postoperative pain. The chosen dosage and administration route adhere to recognised veterinarian protocols for rodent pain treatment post‐orthopaedic surgeries, assuring effective analgesia and limiting potential adverse effects. Only the skin was incised in the sham surgery (*n* = 10).

All mice were kept in conventional laboratory cages at the Experimental Animal Center of Nanfang Hospital. Post‐surgery, each animal was permitted unrestricted weight‐bearing and activity. Animals were provided with a standardised laboratory meal and unrestricted access to water. The housing density was sustained at 3–4 animals per cage to promote their welfare while facilitating typical social interactions. No additional environmental enrichment products were supplied beyond the standard housing configuration.

Figure S1A shows the molecular structure of FD. IVDD mice were treated with FD (0.5 or 3 mg/kg, *n* = 20 per group) dissolved in DMSO, while sham‐operated mice received vehicle (*n* = 10). The treatment schedule is illustrated in Figure S1B.

### 2.3 Cells, Cell Culture and Treatment

NP cells were extracted from the lumbar and caudal intervertebral discs of specified mice after enzymatic digestion using type II collagenase [20]. The extraction procedure for rat primary NP cells is consistent with other research [21]. The extracted NP cells were cultivated in DMEM/F12 (Gibco, USA) supplemented with 10% fetal bovine serum and 1% penicillin‐streptomycin. HEK293T cells were cultured in full DMEM. For pharmacological modulation, cells were pre‐treated with FD at doses of 1 or 4 μM for 8 h; vehicle controls received 0.1% DMSO. HEK293T cells were exposed to IL‐1β (500 ng/mL) or TNF‐α (200 ng/mL) for 6 h in dual‐luciferase reporter assays. To analyse degradation pathways, autophagosome‐ or lysosome‐mediated protein turnover was obstructed with chloroquine (CQ, 50 μM), while proteasome‐mediated degradation was hindered with MG132 (10 μM).

### 2.4 Cell Viability Assay

NP cell viability was assessed using the CCK‐8 assay. Mouse and rat NP cells were seeded into 96‐well plates (5 × 10^3^ cells/cm^2^), allowed to adhere for 18 h and then treated with FD (0–12 μM) for 8 h. Cell viability was determined by measuring absorbance at 450 nm after incubation with 10% CCK‐8 reagent in DMEM, using a microplate reader (Thermo Fisher).

### 2.5 Preparation of Paraffin‐Embedded Specimens, Histochemistry, Immunohistochemistry and Immunostaining

Cervical spine segments from mice were fixed in 4% paraformaldehyde at 4°C for 24 h, decalcified, paraffin‐embedded and sectioned at 4 μm. Safranin‐O/Fast Green staining was used to assess IVDD [22]. NP changes on the cervical vertebra were scored using the Histologic Degeneration Score (HDS) system [23, 24]. For immunofluorescence (IF), sections were subjected to antigen retrieval in 10 mM sodium citrate buffer (pH 6.0), blocked with 10% goat serum for 1 h, incubated with primary antibodies overnight at 4°C and then with Cy3‐conjugated secondary antibodies for 1 h. For immunohistochemistry (IHC), endogenous peroxidase was quenched with 3% H_2_O_2_ for 15 min, followed by blocking with 10% goat serum, overnight incubation with primary antibodies at 4°C and visualisation using DAB with haematoxylin counterstaining.

### 2.6 Luciferase and Reporter Assays

HEK293T cells were seeded in 24‐well plates and transfected with pRL‐TK and the NF‐κB luciferase reporter, with or without additional plasmids. After 18 h, cells were stimulated with LPS (1000 ng/mL), IL‐1β (1000 ng/mL) or TNF‐α (100 ng/mL) for 6–8 h. To examine Vangl2 function, increasing amounts of HA‐Vangl2 plasmid (250, 500 or 1000 ng) or empty vector were co‐transfected. Luciferase activity was measured using the Dual‐Luciferase Reporter Assay Kit on a Luminoskan Ascent luminometer, as described previously [25].

### 2.7 Immunoprecipitation and Immunoblot Analysis

Cells were lysed on ice for 30 min in low‐salt buffer, and lysates were cleared by centrifugation at 14,000× *g* for 15 min at 4°C. For endogenous Co‐IP, precleared lysates were incubated with 2 μg of the indicated antibody or isotype control overnight at 4°C, followed by Protein A/G agarose beads for 4–6 h. Exogenous Co‐IP was performed with anti‐FLAG or anti‐Myc agarose beads for 4 h at 4°C. Immune complexes were washed with ice‐cold buffer and eluted by boiling in 2× Laemmli buffer at 95°C for 8–10 min.

Eluates were resolved by SDS‐PAGE, transferred to 0.22 μm PVDF membranes, blocked with 5% non‐fat milk in TBST for 1 h and incubated with primary antibodies (Table S2) overnight at 4°C, followed by HRP‐conjugated secondary antibodies. Signals were detected using Luminata Western HRP substrate and quantified by densitometry.

### 2.8 Quantitative Real‐Time PCR (qPCR) Analysis

Total RNA was extracted from tissues or cells using TRIzol reagent [26], and cDNA was synthesised with the Starscript II First‐Strand cDNA Synthesis Kit. Quantitative PCR was conducted using 2× RealStar Green Power Mixture on a QuantStudio 6 Flex system, with primer sequences provided in Table S3.

### 2.9 MRI Detection

In vivo MRI exams using the PharmaScan70/16 US (Bruker) were conducted on the mice prior to surgery, then at 5 and 10 weeks postoperatively. All animals were euthanised at 5 and 10 weeks post‐surgery for the evaluation of IVDD. All experimental protocols received approval from the Animal Ethics Committee of Nanfang Hospital, Southern Medical University, and the animals were housed in a controlled environment with regulated temperature and humidity.

### 2.10 NP Explants

NP explants were isolated and maintained under the conditions described above [27]. After 48 h of culture, explants were exposed to IL‐1β (10 ng/mL) for 24 h to induce degenerative changes.

### 2.11 Statistical Analysis

All experiments were performed at least three times independently. Data are presented as mean ± SD. Statistical comparisons between two groups were conducted using a two‐tailed Student's *t*‐test. Curve fitting and additional analyses were performed with GraphPad Prism 8.0 (GraphPad Software, San Diego, CA, USA). *p* < 0.05 was considered statistically significant.

## 3 Results

### 3.1 Friedelin Mitigates IVDD by Reducing NP Degradation

**FIGURE 1:**
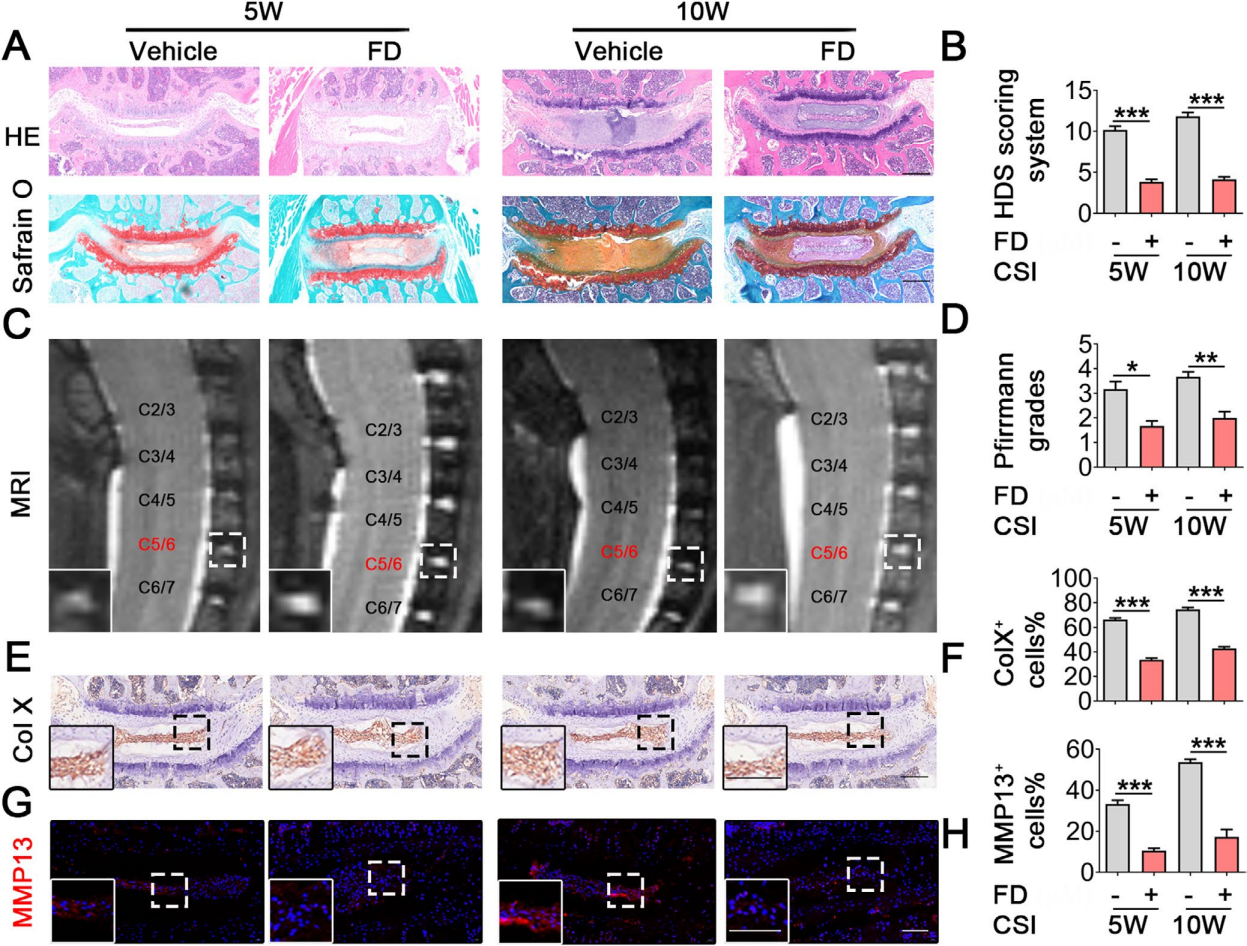
FD alleviates IVDD by decreasing NP degeneration. (A, B) Representative images of HE and Safranin O/Fast Green staining and quantitative analysis of the Histologic Degeneration Score (HDS) in IVDD mice treated with vehicle or 3 mg/kg FD at 5 or 10 weeks post‐surgery. (C, D) Representative images of MRI and quantitative analysis of the Pfirrmann grades were shown. Higher magnification images are shown at the bottom of panel A, with dotted lines indicating the tide line. (E–H) Representative images of IHC staining for Col X, IF for MMP13 and quantitative analysis of positive NP cells in IVDD mice treated with vehicle or FD at 5 or 10 weeks post‐surgery. Scale bars: 50 μm. *n* ≥ 4 per group. Col X, collagen X; CSI, cervical spine instability; FD, Carpaine; IVDD, intervertebral disc degeneration; MMP13, matrix metallopeptidase 13. *p < 0.05, **p < 0.01, ***p < 0.001.

Figure S1A depicts the molecular configuration of FD. To examine the potential influence of FD on the advancement of IVDD, we acquired cervical intervertebral disc tissue specimens from mice undergoing CSI surgery, which were administered either a vehicle or FD treatment (Figure S1B). After evaluating the effects of FD at several doses (0.5 and 3 mg/kg) (Figure S1C), we selected the 3 mg/kg dosage for our following animal investigations. The administration of FD significantly reduced NP degeneration (Figure 1A,B) and resulted in lower Pfirrmann Grades (Figure 1C,D) at both 5 and 10 weeks post‐CSI surgery. Moreover, the biomarkers associated with NP degeneration, specifically Col X and MMP13, demonstrated reduced levels in the FD‐treated group (Figure 1E–H), suggesting that FD alleviated the advancement of IVDD in vivo. Our data collectively indicate that FD mitigates the progression of IVDD by preventing NP degeneration in vivo.

### 3.2 FD Attenuates Inflammatory Responses in NP Cells and In Vivo

Chronic inflammation is a key contributor to IVDD, leading to NP degeneration and fibrous tissue rupture [9]. To investigate the anti‐inflammatory effects of FD, NP cell viability from mice (Figure S2A) and rats (Figure S2B) was first assessed using the CCK‐8 assay, identifying 1 and 4 μM as suitable concentrations.

**FIGURE 2:**
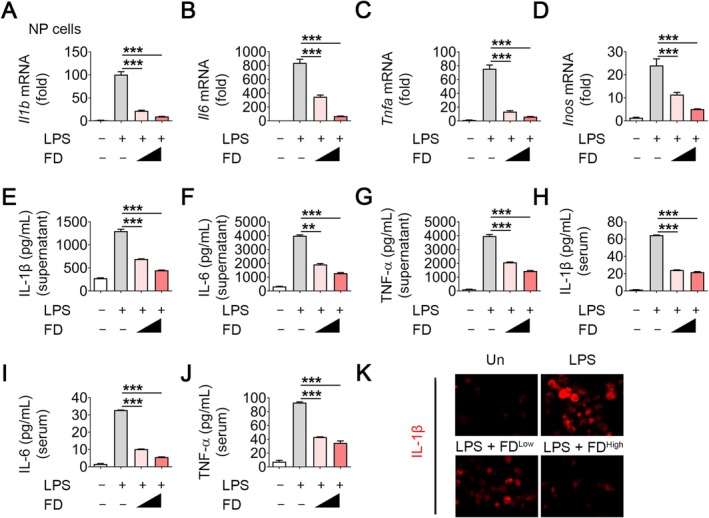
FD decreases inflammatory cytokines in vitro and in vivo. (A–D) Quantitative PCR analysis of *Il1b*, *Il6*, *Tnfa* and *Inos* expression in LPS‐stimulated NP cells treated with or without 1 μM or 4 μM FD. *n* ≥ 4 per group. (E–G) ELISA analysis of IL‐1β, IL‐6 and TNF‐α levels in the supernatant of LPS‐induced NP cells treated with or without 1 μM or 4 μM FD. *n* ≥ 4 per group. (H–J) ELISA analysis of IL‐1β, IL‐6 and TNF‐α levels in the serum of IVDD mice treated with vehicle or FD (0.5 mg/kg or 3 mg/kg) at 5 weeks post‐surgery. *n* ≥ 4 per group. (K) Representative images of immunofluorescence for IL‐1β (red) and quantitative analysis of positive cells in NP of IVDD mice treated with vehicle or 3 mg/kg FD at 5 weeks post‐surgery. *n* ≥ 4 per group. Scale bar: 50 μm. **p < 0.01, ***p < 0.001.

Subsequently, we assessed the protective effects of FD against inflammatory reactions elicited by lipopolysaccharides (LPS) in NP cells. FD treatment attenuated LPS‐induced inflammatory responses in a dose‐dependent manner. In mouse NP cells, FD significantly reduced the expression of IL‐1β, IL‐6, TNF‐α and iNOS at both mRNA (Figure 2A–D) and protein levels in culture supernatants (Figure 2E–G). Similar inhibitory effects were observed in rat NP cells (Figure S2C–I), demonstrating cross‐species efficacy.

Furthermore, in vivo analysis of serum from CSI mice demonstrated that FD treatment lowered systemic levels of IL‐1β, IL‐6 and TNF‐α (Figure 2H–J). These results collectively demonstrate that FD efficiently inhibits NP inflammatory cytokines, underscoring its potential as a treatment agent for IVDD.

### 3.3 Friedelin Mitigates IVDD by Modulating NF‐κB Activity

**FIGURE 3:**
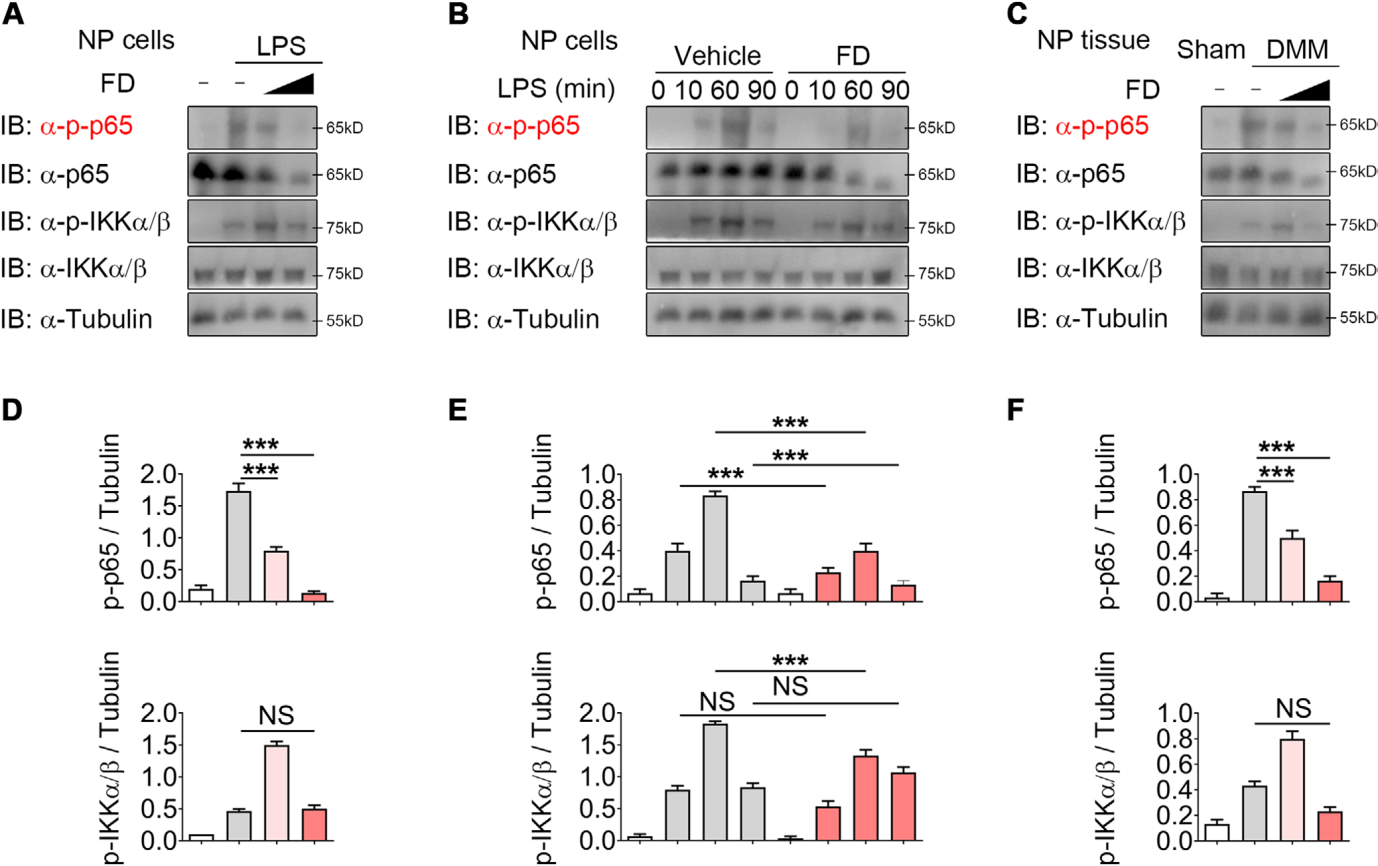
FD inhibits NF‐κB signalling activation during IVDD. (A, D) Western blot analysis and densitometry of p‐p65 and p‐IKKα/β in LPS‐induced NP cells treated with 1 μM or 4 μM FD. *n* = 3 per group. (B, E) Western blot analysis and densitometry of p‐p65 and p‐IKKα/β in LPS‐induced NP cells treated with vehicle or 4 μM FD for various durations. *n* = 3 per group. (C, F) Western blotting and densitometry analysis of p‐p65 and p‐IKKα/β in the NP of IVDD mice treated with or without 0.5 or 3 mg/kg FD. *n* = 3 per group. NS, not significant; ***p < 0.001.

To further clarify the molecular mechanisms underlying FD‐mediated attenuation of IVDD, we investigated its impact on NF‐κB signalling. Previous findings suggest that blocking NF‐κB activity may provide protective benefits against IVDD [10]. In line with this, earlier reports demonstrated that FD alleviates tendinopathy by reducing NF‐κB activation, leading us to propose that FD also hinders IVDD progression via a similar pathway. In our experiments, LPS‐induced NF‐κB activation was tested across multiple FD concentrations in murine NP cells. FD suppressed p65 phosphorylation in a concentration‐dependent fashion, whereas IKK phosphorylation remained largely unaffected (Figure 3A,D). Consistent results were obtained in rat NP cells (Figure S3A,C). Additionally, FD administration reduced p65 phosphorylation following short‐term stimulation (Figure 3B,E; Figure S3B,D), confirming that FD interferes with NF‐κB signalling in vitro. In vivo, NP tissues from CSI mice demonstrated elevated NF‐κB activity 5 weeks post‐surgery. FD treatment selectively decreased p65—but not IKK—phosphorylation, thereby attenuating NF‐κB activation (Figure 3C,F). All of these results suggest that FD slows the progression of IVDD via modifying NF‐κB signalling.

### 3.4 FD Inhibits NF‐κB Signalling via Autophagic Degradation of p65

**FIGURE 4:**
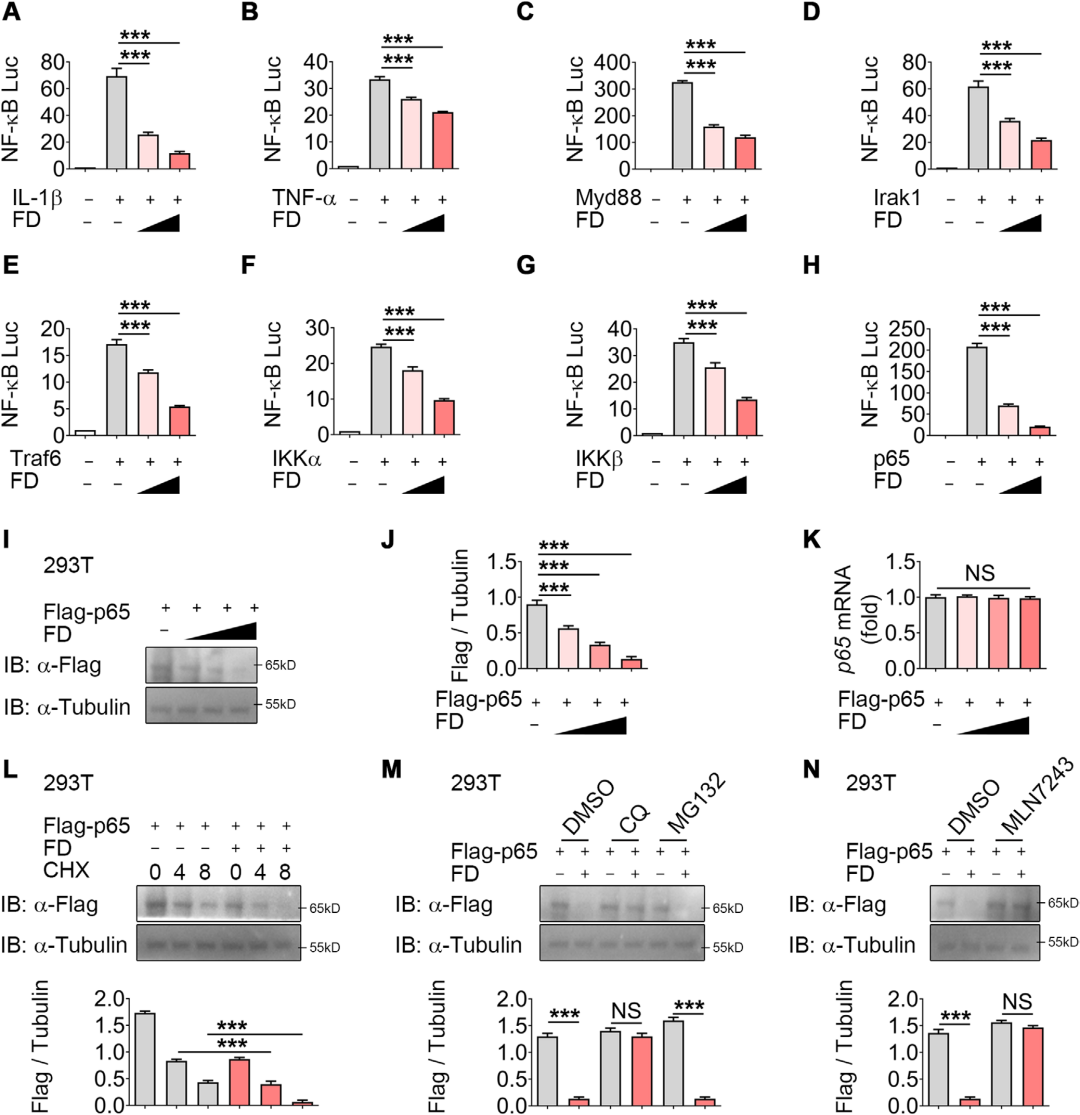
FD suppresses the NF‐κB pathway by promoting ubiquitin‐proteasomal degradation of p65. (A, B) NF‐κB luciferase activity was measured in 293T cells stably expressing the NF‐κB luciferase reporter construct after exposure to IL‐1β or TNF‐α with or without 1 μM or 4 μM FD for 6 h. Luciferase activity is presented as relative light units (RLUs) normalised to Renilla luciferase activity. *n* = 3 per group. (C–H) NF‐κB luciferase activity was measured in 293T cells stably expressing the NF‐κB luciferase reporter construct after overexpression of MyD88, IRAK1, TRAF6, IKKα, IKKβ or p65 with or without 1 μM or 4 μM FD. *n* = 3 per group. (I, J) Western blotting and densitometry analysis of Flag‐p65 in 293T cells transfected with Flag‐p65 and increasing doses of FD for 12 h. *n* = 3 per group. (K) Quantitative PCR analysis of p65 in 293T cells transfected with Flag‐p65 and increasing doses of FD for 12 h. *n* = 3 per group. (L–N) Western blotting and densitometry analysis of Flag‐p65 in 293T cells treated with varying doses of CHX with or without 4 μM FD in indicated times. *n* = 3 per group. (M, N) Western blotting and densitometry analysis of Flag‐p65 in 293T cells treated with DMSO, MG132 (10 μM), CQ (50 μM), 3‐MA (10 mM) or MLN7243 (0.2 μM) for 6 h, with or without 4 μM FD. *n* = 3 per group. NS, not significant; ***p < 0.001.

Using an NF‐κB luciferase reporter in 293T cells, FD dose‐dependently suppressed IL‐1β‐ and TNF‐α–induced NF‐κB activation (Figure 4A,B). To identify its molecular target, MyD88, IRAK1, TRAF6, IKKα, IKKβ or p65 were overexpressed in 293T cells. While each enhanced NF‐κB‐luc activity, FD selectively attenuated their effects, indicating inhibition downstream at the p65 level (Figure 4C–H).

We next examined whether FD directly regulates p65 expression. Transfection of Flag‐tagged p65 into 293T cells, together with increasing doses of FD, revealed a dose‐dependent reduction in p65 protein levels (Figure 4I,J), which was also observed in LPS‐treated murine NP cells (Figure S4A,B). qPCR analysis confirmed that FD did not alter p65 mRNA expression (Figure 4K; Figure S4C), indicating that the decrease occurs at the protein level. Consistently, cycloheximide (CHX)‐chase experiments showed that FD accelerated p65 degradation in both 293T and NP cells (Figure 4L; Figure S4D).

To delineate the mechanism of degradation, 293T cells were transfected with p65 and subsequently exposed to FD together with different pharmacological inhibitors. Chloroquine (CQ), an autolysosome inhibitor, effectively blocked FD‐induced p65 degradation, whereas the proteasome inhibitor MG132 showed no such effect (Figure 4M). Consistent outcomes were observed in LPS‐stimulated NP cells (Figure S4E). Although earlier studies suggested that FD facilitates IL‐1β–driven p65 polyubiquitination and degradation in tendinopathy [15], our data showed that blocking ubiquitination with MLN7243 did not prevent FD‐mediated degradation of p65 (Figure 4N; Figure S4F). These data indicate that FD attenuates NF‐κB activation by facilitating autophagy‐mediated degradation of p65.

### 3.5 FD Promotes p65 Degradation by Recruiting the E3 Ligase RNF182

**FIGURE 5:**
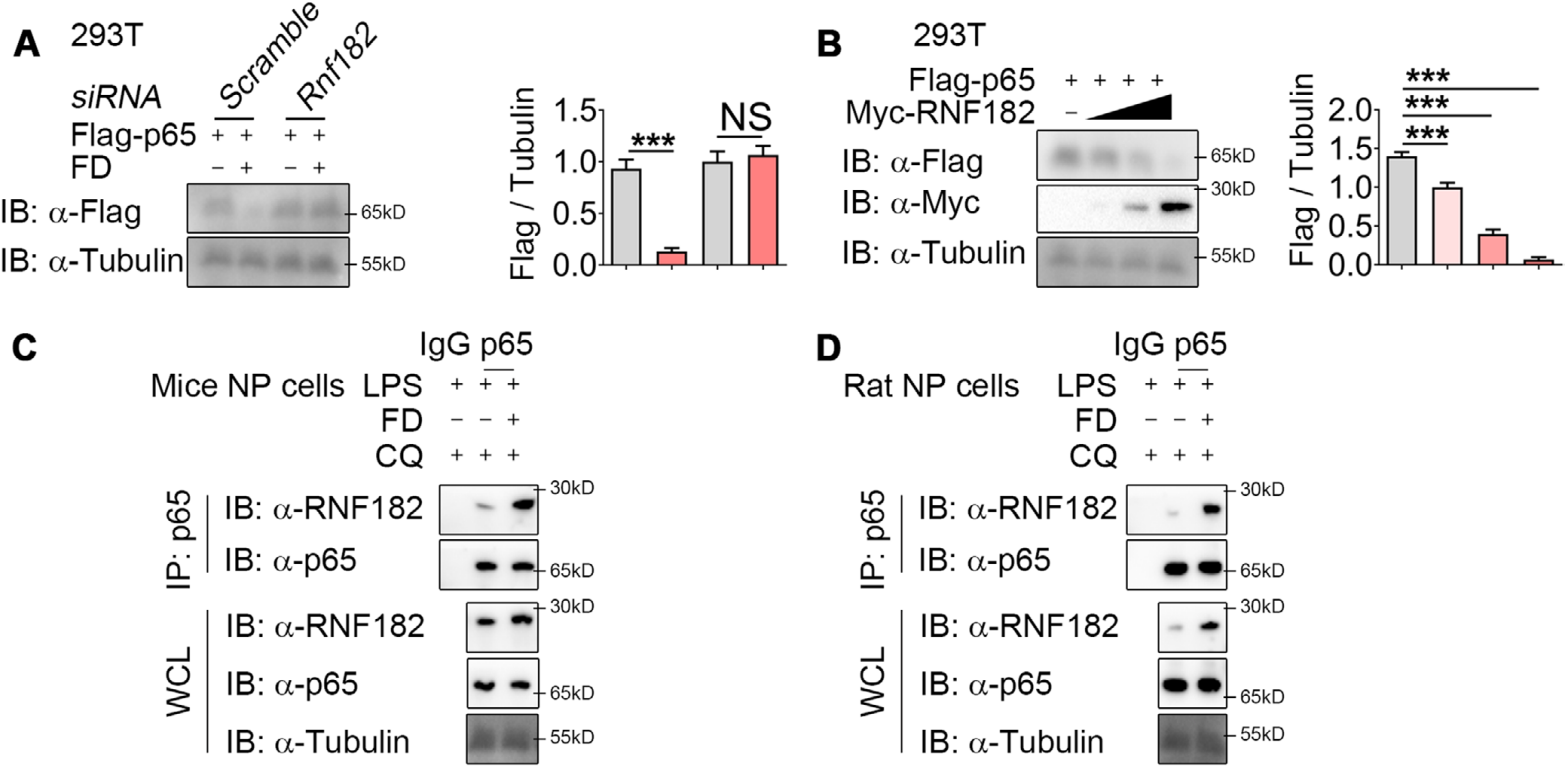
FD enhances the interaction between p65 and RNF182. (A) Western blotting and densitometry analysis of Flag‐p65 in 293T cells transfected with Flag‐p65 and *Rnf182 siRNA*, with or without 4 μM FD for 12 h. *n* = 3 per group. (B) Western blotting and densitometry analysis of Flag‐p65 in 293T cells transfected with Flag‐p65 and varying concentrations of Myc‐RNF182 plasmids for 24 h. *n* = 3 per group. (C, D) Immunoprecipitation of p65 from 293T cells pre‐treated with MG132 (10 μM) for 6 h, followed by stimulation with LPS or Myc‐RNF182 plasmids and FD for 24 h. Western blot analysis was performed to detect the protein levels of p65 and RNF182 in both immunoprecipitated (IP) and whole cell lysate (WCL) samples. NS, not significant; ***p < 0.001.

E3 ubiquitin ligases are essential for the regulation of protein ubiquitination and degradation. Using the prediction tool UbiBrowser (http://ubibrowser.bio‐it.cn/ubibrowser_v3/home/index), we identified RNF182, MKRN2 and LRSAM1 as potential E3 ligases targeting p65. Following FD treatment, RNF182 mRNA expression was altered, whereas MKRN2 and LRSAM1 remained unchanged (Figure S5A). Previous work has shown that FD promotes p65 degradation through RNF182 in tendinopathy [15]. Consistent with previous findings in tendinopathy, silencing RNF182 abolished the protective effect of FD (Figure 5A), whereas RNF182 overexpression induced dose‐dependent p65 degradation (Figure 5B).

We speculated that FD could facilitate the association of RNF182 with p65. Co‐immunoprecipitation analysis confirmed that FD strengthened the interaction between these two proteins (Figure 5C,D). Moreover, the inhibition of RNF182 activity eliminated FD‐induced ubiquitination of p65 (Figure S5B). Collectively, these findings indicate that FD expedites p65 degradation via enlisting the E3 ubiquitin ligase RNF182.

### 3.6 FD Mitigates IVDD via Enlisting RNF182

**FIGURE 6:**
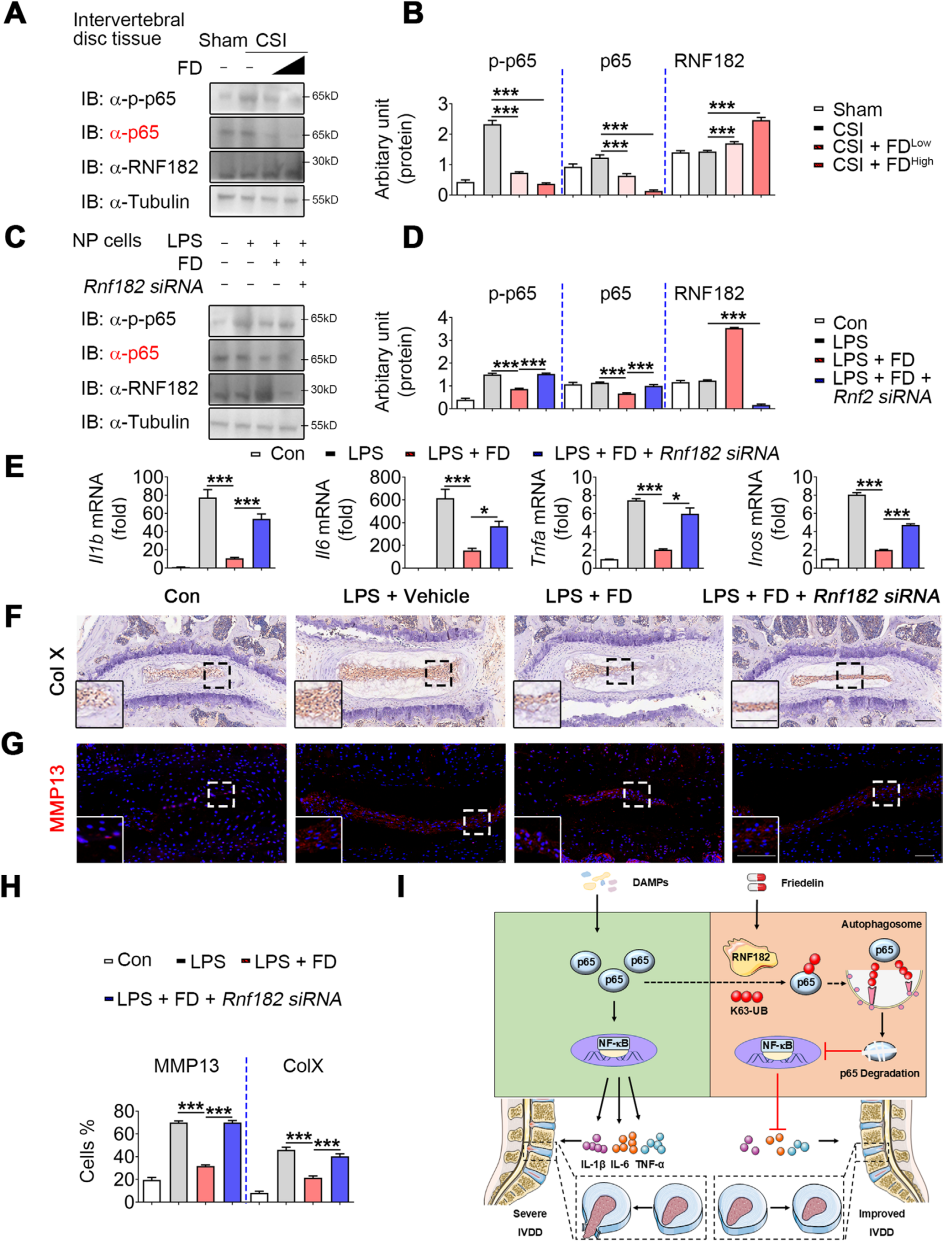
FD inhibits the NF‐κB pathway by recruiting RNF182 to degrade p65 during IVDD. Mice underwent sham surgery plus saline (sham), CSI plus saline (CSI), CSI plus 0.5 μM FD (CSI+FD^low^) or CSI plus 3 μM FD (CSI+FD^high^) (i.p., *n* = 3 each). NP explants were treated with vehicle (Con) and LPS‐induced NP explants were treated with vehicle (LPS+vehicle), FD (LPS+FD) or FD plus Rnf182‐siRNA (LPS+FD+siRnf182). (A, B) Western blotting and densitometry analysis of p‐p65, p65 and RNF182 in NP from IVDD mice treated with or without 0.5 μM or 3 μM FD. *n* = 3 per group. (C, D) Western blotting and densitometry analysis of p‐p65, p65 and RNF182 in NP cells induced by LPS and treated with or without FD and *Rnf182 siRNA*. (E) Quantitative PCR analysis of *Il1b*, *Il6*, *Tnfa* and *Inos* in samples described in (C, D). *n* = 3 per group. (F–H) Representative images of IHC staining for Col X, immunofluorescence for MMP13 and quantitative analysis of positive NP cells in IVDD mice treated with or without FD and *Rnf182 siRNA*. Scale bars: 50 μm. *n* ≥ 4 per group. (I) FD decreases NP inflammation and inhibits NF‐κB signalling by recruiting RNF182 to promote ubiquitin‐proteasomal degradation of p65. NS, not significant; *p < 0.05, ***p < 0.001.

The above findings suggest that FD suppresses NF‐κB activation through RNF182. To further evaluate the protective role of RNF182 in IVDD, we examined its expression in disc tissues of CSI mice with or without FD treatment. RNF182 levels showed no difference between Sham and CSI groups; however, FD administration markedly increased RNF182 expression compared with vehicle controls (Figure 6A,B).

To verify whether RNF182 mediates FD‐induced anti‐inflammatory effects in NP cells, we silenced Rnf182 using siRNA in LPS‐stimulated murine NP cells. Knockdown of RNF182 abolished the ability of FD to suppress p65 phosphorylation and degradation (Figure 6C,D). Consistently, the reduction in pro‐inflammatory mediators (*Il1b, Il6, Tnfa* and *Inos*) by FD was largely reversed upon RNF182 depletion (Figure 6E), with similar results observed in cell supernatants (Figure S6A). These findings indicate that FD attenuates NF‐κB activation and cytokine production through RNF182 in IVDD.

We next investigated whether FD protects against IVDD progression and NP degradation via RNF182. NP explants cultured with LPS, together with Rnf182 siRNA, demonstrated that RNF182 knockdown impaired the protective effect of FD. Specifically, FD‐mediated suppression of Col X and MMP13 expression was reversed by RNF182 silencing (Figure 6F,G). Similarly, in LPS‐treated NP cells, RNF182 inhibition increased Col10a1 and Mmp13 expression, while decreasing Col2a1 and Sox9 levels (Figure S6B). Together, these results demonstrate that FD alleviates NP cell catabolism and disc degeneration through RNF182‐dependent anti‐inflammatory mechanisms.

## 4 Discussion

IVDD is a prevalent condition that considerably affects spinal health and overall quality of life [28, 29]. It is marked by increasing structural degradation and functional impairment of the intervertebral disc, often resulting in chronic low back pain and disability [30, 31]. The process of degeneration is closely linked to inflammatory responses, which are crucial in the development of IVDD [32, 33]. Chronic inflammation significantly contributes to the degeneration of the nucleus pulposus, the major component of the intervertebral disc, resulting in altered biomechanics and increased susceptibility to further injury [34, 35]. Consequently, clarifying the molecular processes of IVDD is essential for creating effective treatments to mitigate this illness and enhance patient quality of life.

This study investigates the possible preventive effects of FD, a natural triterpenoid, against IVDD by analysing its anti‐inflammatory properties and its influence on the NF‐κB signalling system. Our findings demonstrate that FD substantially mitigates NP degradation and reduces pro‐inflammatory cytokine production by regulating NF‐κB activation. FD specifically promotes the autophagic breakdown of p65, which helps to reduce the inflammation associated with IVDD. This research offers valuable insights into the therapeutic potential of FD as an anti‐inflammatory agent for IVDD, setting the stage for further studies and clinical applications.

This study demonstrates that FD protects against IVDD by modulating NF‐κB signalling through autophagy‐mediated degradation of p65. This procedure diminishes pro‐inflammatory cytokines and aids in mitigating NP degradation. This discovery is particularly important as it introduces a new therapeutic approach for managing IVDD by focusing on autophagy, a cellular process recognised for its role in maintaining cellular balance and regulating inflammatory responses. Additionally, FD selectively inhibits the phosphorylation of p65 without impacting IKK phosphorylation, indicating a precise regulatory mechanism that could be utilised in developing targeted therapies for inflammatory diseases associated with abnormal NF‐κB activation.

While E3 ubiquitin ligases play established roles in IVDD, their specific involvement in regulating the p65 subunit of NF‐κB remains virtually unexplored. Current research has primarily examined E3 ligases such as Parkin in mitophagy‐mediated mitochondrial clearance 1 and ITCH (regulated by hsa_circ_0059955) in apoptosis control 2 [36, 37, 38] yet no studies have addressed p65‐targeting E3 ligases within disc biology. Despite the role of multiple E3 ubiquitin ligases [39] (e.g., Pdlim7, RNF182, FBXW7, FBXW4, TRIM7, TRIM21, SOCS1, FBOX6, FBXO7, MKRN2, LRSAM1, FBXW8) in regulating p65 stability across inflammatory disorders, their functions in IVDD remain largely unexplored [40, 41]. Our study found that the natural triterpenoid FD specifically recruits RNF182 to trigger autophagic degradation of p65 in NP cells. Mechanistically, Friedelin: (1) upregulates RNF182 expression, (2) promotes RNF182‐p65 co‐immunoprecipitation and (3) induces K48‐linked polyubiquitination of p65, leading to its autophagy degradation rather than proteasomal clearance. Crucially, RNF182 knockdown abolished FD‐mediated p65 reduction and nullified anti‐inflammatory effects. These findings establish a novel proteostasis mechanism diverging from known E3 ligase functions in IVDD: distinct from mitophagy regulation by Parkin 1 or apoptosis control by ITCH2. Our data demonstrate that RNF182 functions as the first identified E3 ubiquitin ligase directly targeting p65 in disc cells to mediate its autophagic degradation, thereby establishing its potential as a key regulator of NF‐κB signalling in IVDD.

Furthermore, our findings elucidate FD's impact beyond molecular interactions, highlighting its regulatory effects on key cellular functions and genomic activity. FD treatment markedly reduced expression of pro‐inflammatory cytokines (IL‐1β, TNF‐α), demonstrating its dual role in inhibiting acute inflammatory responses and facilitating prolonged adaptive processes linked to tissue repair. This delineation of NP cell responses advances our comprehension of inflammatory adaptation and establishes FD as a probe for exploring genomic regulation underlying chronic degeneration and potential regenerative pathways.

Moreover, this research underscores FD's immunological potential as an anti‐inflammatory agent for IVDD and related disorders. By elucidating its mechanism in regulating NF‐κB‐dependent immune responses, the findings demonstrate FD's dual action: mitigating IVDD symptoms and targeting the underlying inflammatory drivers of disease progression. These insights align with growing interest in natural compounds as complementary therapies for chronic inflammation. Consequently, our work provides a comprehensive understanding of FD's therapeutic mechanism—modulating both autophagic degradation and NF‐κB signalling—highlighting its promise for IVDD treatment. Further investigation into FD's pharmacology is warranted to translate this natural compound into novel therapeutic strategies for inflammatory and degenerative diseases. This study has important limitations that warrant consideration, including the lack of clinical validation and limited animal cohort sizes. Furthermore, assessing potential long‐term effects and drug interactions is essential to fully evaluate FD's therapeutic efficacy and safety.

In conclusion, our findings demonstrate that FD inhibits NF‐κB signalling by facilitating RNF182‐mediated autophagic degradation of p65, thereby suppressing pro‐inflammatory cytokine production and ameliorating IVDD. This mechanism, focused on FD's recruitment of the E3 ubiquitin ligase RNF182, positions FD as a viable target for IVDD therapy. Additional examination of FD in clinical environments is necessary to convert these discoveries into improved therapeutic approaches for this inflammatory illness.

## Author Contributions

All authors have made a substantial contribution to the study and take public responsibility for their specific roles as listed below. Design: Qiang Wu, Xiangheng Dai. Data acquisition: Kewu Tu, Zhaomou Chen, Dongteng Liao, Zhenyu Wang, Hongyu Zhong, Xiangheng Dai. Analysis and interpretation of data: Kun Zhao, Shimin Wu, Huiyin Zhu, Jinming Xu, Beidi Zhou. All authors read and approved the final manuscript.

## Funding

This work was also supported by the 2022 Shaoguan City Science and Technology Bureau Shaoguan City Social Development Science and Technology Collaborative Innovation System Construction Project (High‐level Hospital Construction Research Project) (220602184532348).

## Conflicts of Interest

The authors declare no conflicts of interest.

## Supporting information


**Table S1:** The reagents involved in this study.
**Table S2:** The antibodies involved in this study.
**Table S3:** Primers for real‐time RT‐PCR used in this study.
**Figure S1:** Optimal FD dose screening in IVDD mice. (A) The molecular structure of FD was demonstrated. (B) Diagram of experiment procedure is shown. 10‐week‐old mice were subjected to IVDD surgery and treated with vehicle or FD at day 7 (1 time/week), cervical spine were collected at 5 or 10 weeks after IVDD surgery. (C) Safranin O and Fast Green staining sagittal views of intervertebral disc at 5 weeks after IVDD surgery. Dotted lines demonstrate tide line. Sham, IVDD treated with vehicle, IVDD treated with 0.5 or 3 mg/kg FD. Scale bar: 50 μm. IVDD, destabilising the medial meniscus; FD, Carpaine; Sham, sham‐surgery.
**Figure S2:** FD effects on cell viability in NP cells from mice or rats. (A, B) Mice NP cells (A) and rat NP cells (B) were cultured with diverse concentrations of FD (0–12 μM) for 12 h, and cell viability was analysed by Cell Counting Kit‐8 assay. (C–I) LPS‐induced rat NP cells were treated with diverse concentrations of FD for 12 h. Quantitative PCR analysis of *Il1b*, *Il6*, *Tnfa* and *Inos* in LPS‐induced rat NP cells treated with or without FD (C–I). Supernatants were collected and subjected to ELISA analysis of IL‐1β, IL‐6 and TNF‐α expression (*n* ≥ 4) (G–I). NS, not significant; **p < 0.01, ***p < 0.001.
**Figure S3:** Carpaine prevents NF‐κB activation in rat NP cells. (A, B) Western bloting analysed the NF‐κB pathway‐related proteins (p‐IKK, IKK, p‐p65 and p65) in LPS‐induced rat NP cells treated with diverse concentrations of FD (1 and 4 μM) (A) or with 4 μM FD at a different time (0–60 min) (B). (C, D) Analysis of grey intensity was shown in A and B. NS, not significant; ***p < 0.001.
**Figure S4:** FD exerts its protective effect on the NF‐κB pathway by facilitating the ubiquitin‐proteasomal degradation of p65. (A–C) Following LPS treatment for 6 h, NP cells were exposed to FD (1, 2 and 4 μM) for 6 h and then the detection of p65 protein was detected by Western blotting method (A). Analysis of grey intensity was shown in A (B). p65 mRNA expression was detected by using PCR method (C). (D) NP cells were treated with CHX for the indicated times, and the expression of p65 was detected by immunoblot. Analysis of grey intensity was shown in D. (E) NP cells were induced by LPS, administrated with vehicle or FD and treated with DMSO, MG132 (10 μM), CQ (50 μM) for 6 h. The cell lysates were analysed by immunoblot. Analysis of grey intensity was shown in E. (F) LPS‐induced BMDMs were administrated with vehicle or FD and treated with or without MLN7243, and the expression of p65 was detected by immunoblot. Analysis of grey intensity was shown in F. NS, not significant; ***p < 0.001.
**Figure S5:** FD increases the interaction p65 and RNF182 and increases the polyubiquitination of p65. (A) LPS‐induced mice NP cells were treated with diverse concentrations of FD for 12 h. Quantitative PCR analysis of *Rnf18, Mkrn2, Lrsam1* in LPS‐induced rat NP cells treated with or without FD. (B) NP cells underwent Lrsam1 siRNA silencing for 24 h. And the cells were stimulated with CQ (50 μM) for 6 h and exposed to LPS (100 ng/mL) and FD (4 μM) for 8 h. The cell lysates were subjected to immunoprecipitation with an anti‐p65 antibody or control IgG and immunoblotted with the indicated antibodies. NS, not significant; ***p < 0.001.
**Figure S6:** FD reduces inflammation and NP destruction by recruiting RNF182. (A) ELISA analysis of IL‐1β, IL‐6 and TNF‐α levels in the supernatant of LPS‐induced NP cells treated with vehicle, FD or FD plus *Lrsam1 siRNA*. (B) NP cells were co‐cultured with CM, vehicle‐treated M1 macrophage CM, FD‐treated NP cells CM or FD plus *Lrsam1 siRNA*‐treated NP cells CM for 24 h. Quantitative PCR analysis of Col10a1, MMP13, Col2a1 and Sox9 in NP cells. ***p < 0.001.

## Data Availability

The data that supports the findings of this study are available in the Supporting Information of this article.
