# Supplementary material for: Friedelin Ameliorates Nucleus Pulposus Inflammation by Increasing p65 Autophagic Degradation to Inhibit NF‐κB Signalling Pathway
Source: J Cell Mol Med. 2026 Jan 8;30(1):e70989. doi: 10.1111/jcmm.70989 (PMC12780968; doi:10.1111/jcmm.70989)
Supplement: Supplementary file 1 — Table S1: The reagents involved in this study. Table S2: The antibodies involved in this study. Table S3: Primers for real‐time RT‐PCR used in this study. Figure S1: Optimal FD dose screening in IVDD mice. (A) The molecular structure of FD was demonstrated. (B) Diagram of experiment procedure is shown. 10‐week‐old mice were subjected to IVDD surgery and treated with vehicle or FD at day 7 (1 time/week), cervical spine were collected at 5 or 10 weeks after IVDD surgery. (C) Safranin O and Fast Green staining sagittal views of intervertebral disc at 5 weeks after IVDD surgery. Dotted lines demonstrate tide line. Sham, IVDD treated with vehicle, IVDD treated with 0.5 or 3 mg/kg FD. Scale bar: 50 μm. IVDD, destabilising the medial meniscus; FD, Carpaine; Sham, sham‐surgery. Figure S2: FD effects on cell viability in NP cells from mice or rats. (A, B) Mice NP cells (A) and rat NP cells (B) were cultured with diverse concentrations of FD (0–12 μM) for 12 h, and cell viability was analysed by Cell Counting Kit‐8 assay. (C–I) LPS‐induced rat NP cells were treated with diverse concentrations of FD for 12 h. Quantitative PCR analysis of Il1b, Il6, Tnfa and Inos in LPS‐induced rat NP cells treated with or without FD (C–I). Supernatants were collected and subjected to ELISA analysis of IL‐1β, IL‐6 and TNF‐α expression (n ≥ 4) (G–I). NS, not significant; **p < 0.01, ***p < 0.001. Figure S3: Carpaine prevents NF‐κB activation in rat NP cells. (A, B) Western bloting analysed the NF‐κB pathway‐related proteins (p‐IKK, IKK, p‐p65 and p65) in LPS‐induced rat NP cells treated with diverse concentrations of FD (1 and 4 μM) (A) or with 4 μM FD at a different time (0–60 min) (B). (C, D) Analysis of grey intensity was shown in A and B. NS, not significant; ***p < 0.001. Figure S4: FD exerts its protective effect on the NF‐κB pathway by facilitating the ubiquitin‐proteasomal degradation of p65. (A–C) Following LPS treatment for 6 h, NP cells were exposed to FD (1, 2 and 4 μM) for 6 h and t [file JCMM-30-e70989-s001.docx]

**Friedelin ameliorates nucleus pulposus inflammation by increasing p65 autophagic degradation to inhibit NF-κB Signaling Pathway**

**Additional files1**

**Supplementary Materials and Methods**

**Primary NP culture**

NP were isolated from 4-weeks-old mice. NP tissue were isolated from 4-weeks-old mice tail intervertebral discs and digested with Type II collagenase. The isolated NP cells were cultured in DMEM/F12 medium with fetal bovine serum and penicillin-streptomycin. Second or third passage cells were used for experiments. Explants cultured in DMEM/F12 were treated with 10 mM PBS for 1 h, followed by treatment with LPS, LPS + FD or LPS + FD + *Rnf182 siRNA* for 72 h. The explants were harvested and the medium was collected after 3 days culture.

**ELISA**

Cell supernatants and serum samples were analyzed using mouse IL-1β, IL-6, and TNF-α ELISA kits. Absorbance was measured at 450 nm using the Multiscan FC.

**Supplementary Table**

**Table S1** shows the reagents involved in this study.

| **Reagents** | **Source** | **Identifier** |
| --- | --- | --- |
| Cell Counting Kit-8 | Beyotime | C0037 |
| DMSO | Sigma-Aldrich | D2650 |
| DMEM | KeyGEN | KGL1206 |
| DMEM/F12 | KeyGEN | KGL1201 |
| RPMI 1640 | KeyGEN | KGL1501 |
| FBS | KeyGEN | KGA6008 |
| Recombinant TNFα | Biovision | 1051-1000 |
| ITS | Beyotime | C0343 |
| M-CSF | Sigma-Aldrich | M9170 |
| Recombinant IL-1β | Abcam | ab9723 |
| 3-methyladenine | Sigma-Aldrich | 5142-23-4 |
| MLN7243 | Sigma-Aldrich | HY-100487 |
| MG-132 | Sigma-Aldrich | C-2211 |
| Cycloheximide | Sigma-Aldrich | C7698 |
| Chloroquine | Glpbio | 1954/5/7 |
| LPS | Sigma-Aldrich | L2630 |
| proteinase K | Sigma-Aldrich | 39450-01-6 |
| Hematoxylin and eosin dyes | Beyotime | C0105S |
| Protein G beads | GenScript | L00209 |
| Lipofectamine 2000 | ThermoFisher | 11668019 |
| TRIZOL | Beyotime | R0016 |
| Trypsinization | Gibco | R001100 |
| Amphotericin B | Gibco | 15290026 |
| Penicillin/streptomycin | Gibco | 15140122 |
| PVDF membranes | Sigma-Aldrich | IPVH85R |
| Real-Time PCR Mix | Vazyme | Q331 |
| isoflurane | RWD | R510-22 |
| Dapi | Beyotime | C1006 |
| Type II collagenase | Proteintech | 28459-1-AP |

Table S2 shows the antibodies involved in this study.

| Antibodies | Source | Identifier |
| --- | --- | --- |
| Anti-MMP13 | Proteintech | 83188-4-RR |
| Anti-p-IKK | Cell Signaling Technology | #2697 |
| Anti-IKK | Cell Signaling Technology | #2678 |
| Anti-ColX | Proteintech | 26984-1-AP |
| Anti-p-p65 | Cell Signaling Technology | #3039 |
| Anti-p65 | Santa Cruz Biotechnology | sc-8008 |
| Anti-Flag agarose gels | Sigma-Aldrich | A2220 |
| Anti-Myc | Beijing Ray Antibody Biotech | RM1003 |
| Anti-HA | Roche Applied Science | 12013819001 |
| Anti-Ub | Santa Cruz Biotechnology | sc-8017 |
| Anti-Tubulin | Proteintech | 66200-1-Ig |
| Anti-RNF182 | Abcam | ab246935 |
| Horseradish peroxidase (HRP)-anti-Flag | Sigma-Aldrich | A8592 |
| Goat anti-rabbit IgG (H&L) | Beijing Ray Antibody Biotech | RM3002 |
| Anti-RNF182 | Cell signaling technology | #28405 |
| Goat anti-mouse IgG (H&L) | Beijing Ray Antibody Biotech | RM3001 |
| Goat anti-rabbit IgG (H&L) Alexa Fluor 594 | Immunoway | RS23420 |
| Goat anti-rabbit IgG (H&L) Alexa Fluor 488 | Immunoway | RS23220 |
|  |  |  |

**Table S3: Primers for real-time RT-PCR used in this study.**

| **Gene** | **Sequences** | **Species** |
| --- | --- | --- |
| *Col2a1* | Forward: CACACTGGTAAGTGGGGCAAGACCG  Reverse: GGATTGTGTTGTTTCAGGGTTCGGG | Mouse |
| *Sox9* | Forward: TACCTACGGCATCAGCAGCTC  Reverse: TTGCCTTCACGTGGCTTTAAG | Mouse |
| *Il6* | Forward: CTCTGGGAAATCGTGGAAAT  Reverse: CCAGTTTGGTAGCATCCATC | Mouse |
| *Il1b* | Forward: GCAACTGTTCCTGAACTCAACT  Reverse: GTGCTCATGTCCTCATCCTG | Mouse |
| *Tnfa* | Forward: GACGTGGAACTGGCAGAAGAG  Reverse: TTGGTGGTTTGTGAGTGTGAG | Mouse |
| *Col10a1* | Forward: AAAGCTTACCCAGCAGTAGG  Reverse: ACGTACTCAGAGGAGTAGAG | Mouse |
| *Mmp13* | Forward: CTTCTTCTTGTTGAGCTGGACTC  Reverse: CTGTGGAGGTCACTGTAGACT | Mouse |
| *Gapdh* | Forward: AGGTCGGTGTGAACGGATTTG  Reverse: TGTAGACCATGTAGTTGAGGTCA | Mouse |

|  |  |  |
| --- | --- | --- |

**Supplementary figures**


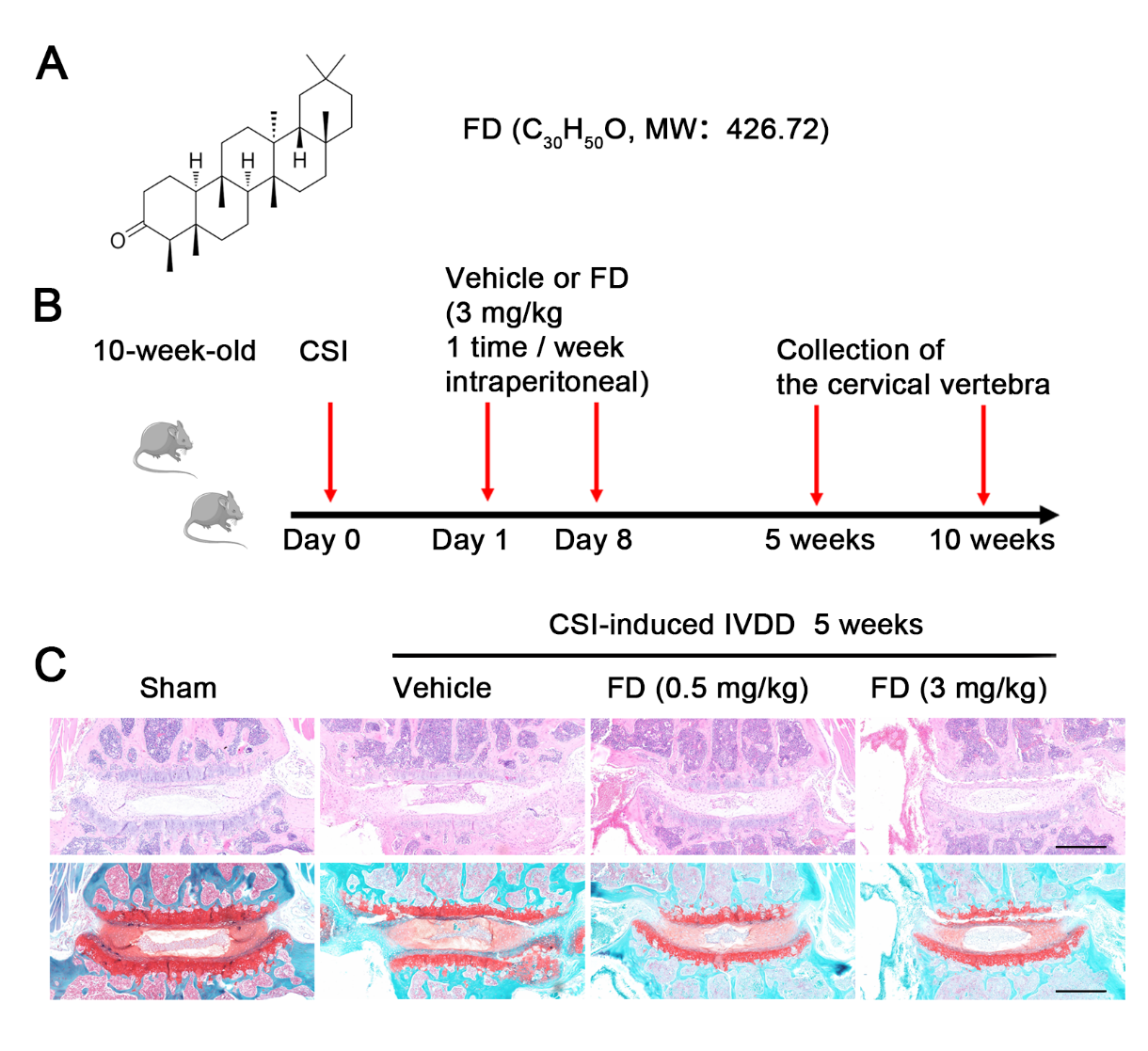


**Figure S1.** Optimal FD dose screening in IVDD mice. (A) The molecular structure of FD was demonstrated. (B) Diagram of experiment procedure is shown. 10-week-old mice were subjected to IVDD surgery, and treated with vehicle or FD at day 7 (1 time / week), cervical spine were collected at 5 or 10 weeks after IVDD surgery. (C) Safranin O and Fast Green staining sagittal views of intervertebral disc at 5 weeks after IVDD surgery. Dotted lines demonstrate tide line. Sham, IVDD treated with vehicle, IVDD treated with 0.5 or 3 mg/kg FD. Scale bar: 50 µm. IVDD, destabilizing the medial meniscus; FD, Carpaine; Sham, sham-surgery.


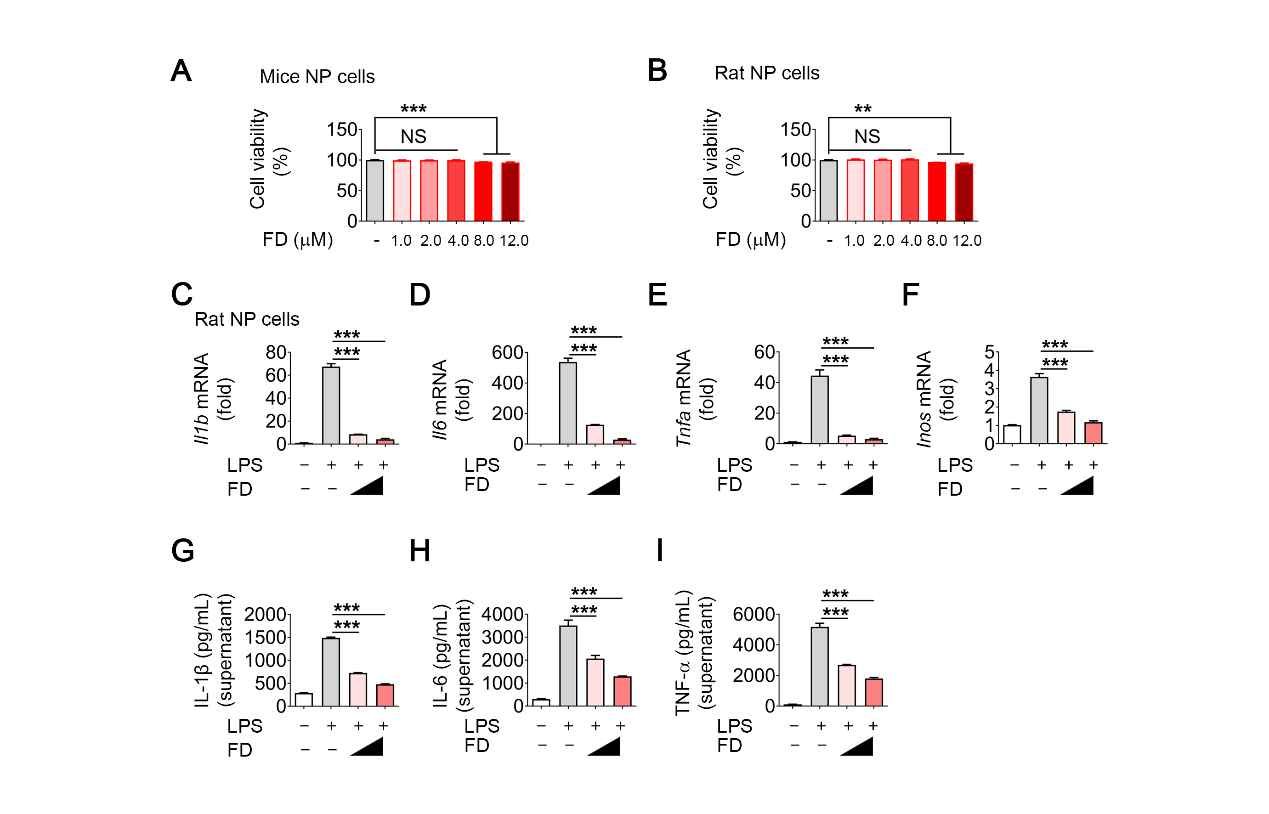


**Figure S2.** FD effects on cell viability in NP cells from mice or rats. (A and B) Mice NP cells (A) and rat NP cells (B) were cultured with diverse concentrations of FD (0–12 µM) for 12 h, and cell viability was analyzed by Cell Counting Kit-8 assay. (C-I) LPS-induced rat NP cells were treated with diverse concentrations of FD for 12 h. Quantitative PCR analysis of *Il1b*, *Il6*, *Tnfa* and *Inos* in LPS-induced rat NP cells treated with or without FD (C-I). Supernatants were collected and subjected to ELISA analysis of IL-1β, IL-6 and TNF-α expression (n≥4) (G-I). NS, not significant; ***p*<0.01, ****p*<0.001.


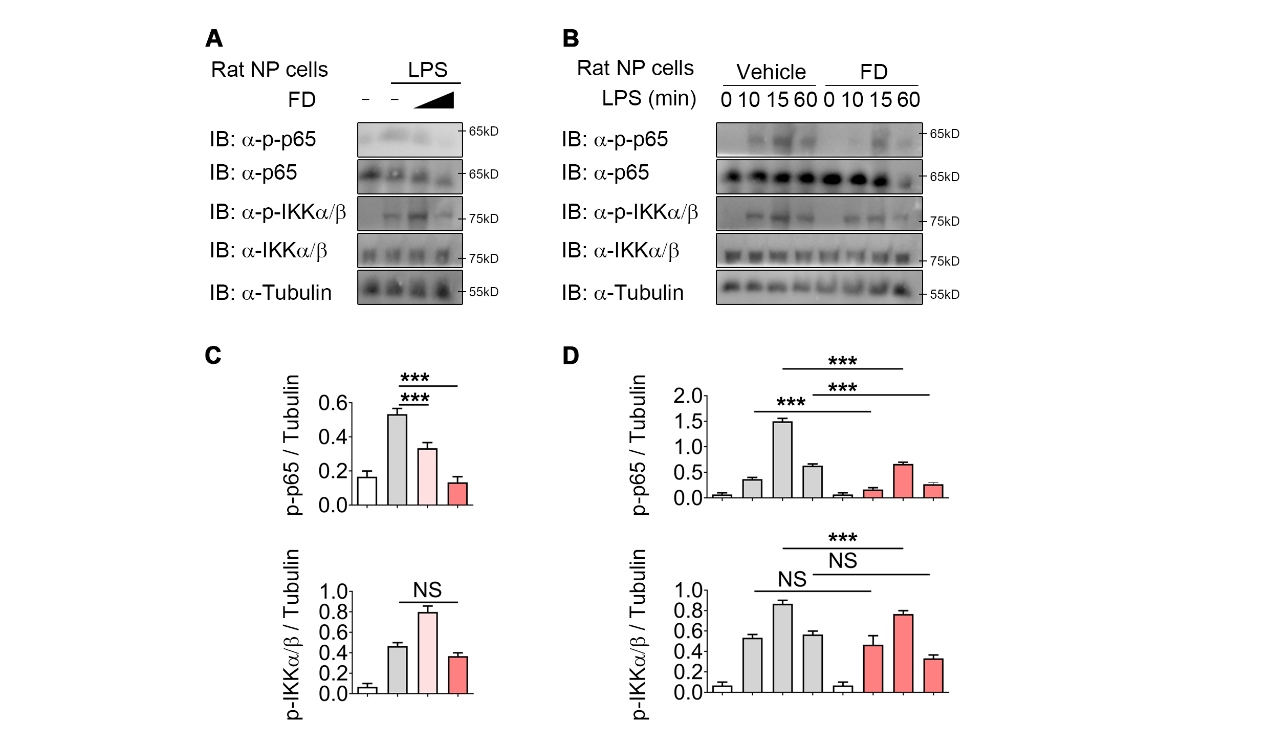


**Figure S3.** Carpaine prevents NF-κB activation in rat NP cells. (A and B) Western bloting analyzed the NF-κB pathway-related proteins (p-IKK, IKK, p-p65, and p65) in LPS-induced rat NP cells treated with diverse concentrations of FD (1 and 4 μM) (A), or with 4 μM FD at a different time (0–60 min) (B). (C and D) Analysis of grey intensity was shown in A and B. NS, not significant; ****p*<0.001.


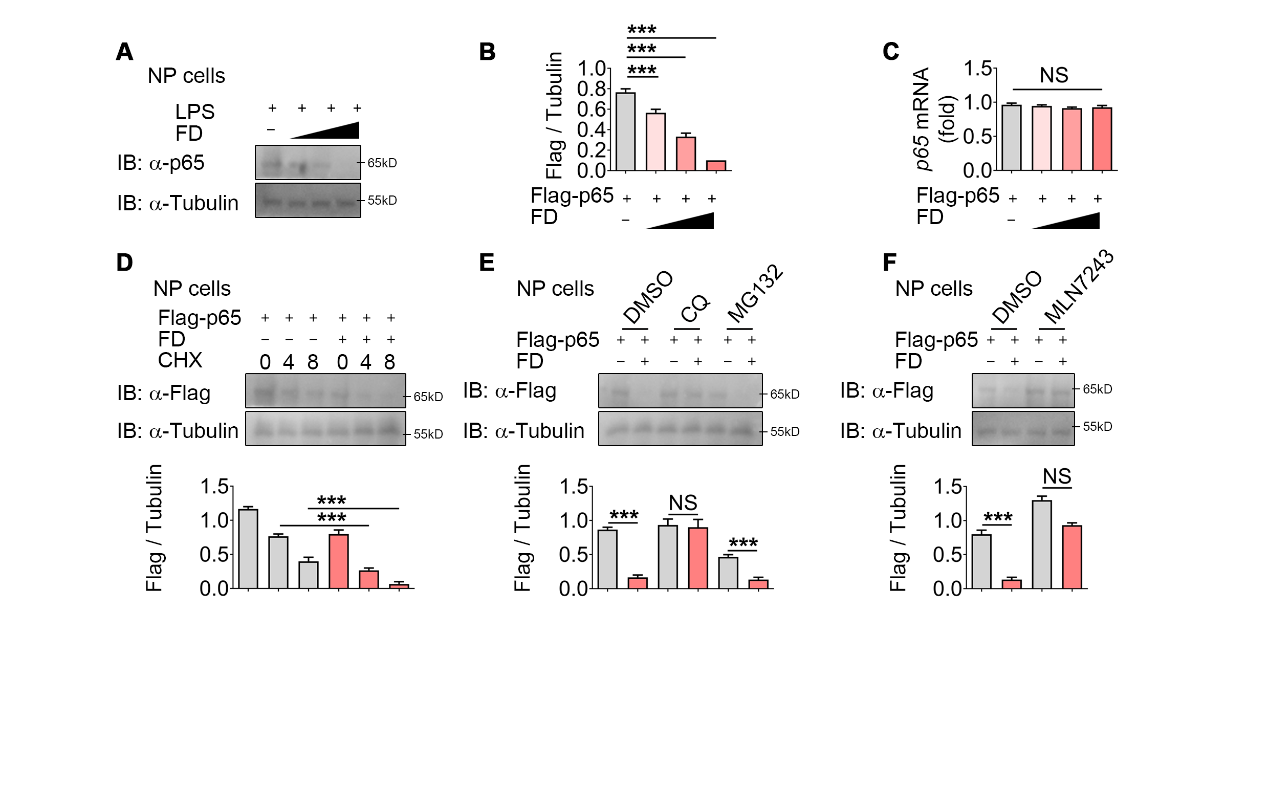


**Figure S4.** FD exerts its protective effect on the NF-κB pathway by facilitating the ubiquitin-proteasomal degradation of p65. (A-C) Following LPS treatment for 6 h, NP cells were exposed to FD (1, 2 and 4 μM) for 6 h, and then the detection of p65 protein was detected by Western blotting method (A). Analysis of grey intensity was shown in A (B). p65 mRNA expression was detected by using PCR method (C). (D) NP cells were treated with CHX for the indicated times, and the expression of p65 was detected by immunoblot. Analysis of grey intensity was shown in D. (E) NP cells were induced by LPS, administrated with vehicle or FD, and treated with DMSO, MG132 (10 μM), CQ (50 μM) for 6 h. The cell lysates were analyzed by immunoblot. Analysis of grey intensity was shown in E. (F) LPS-induced BMDMs were administrated with vehicle or FD, and treated with or without MLN7243, and the expression of p65 was detected by immunoblot. Analysis of grey intensity was shown in F. NS, not significant; ***p<0.001.


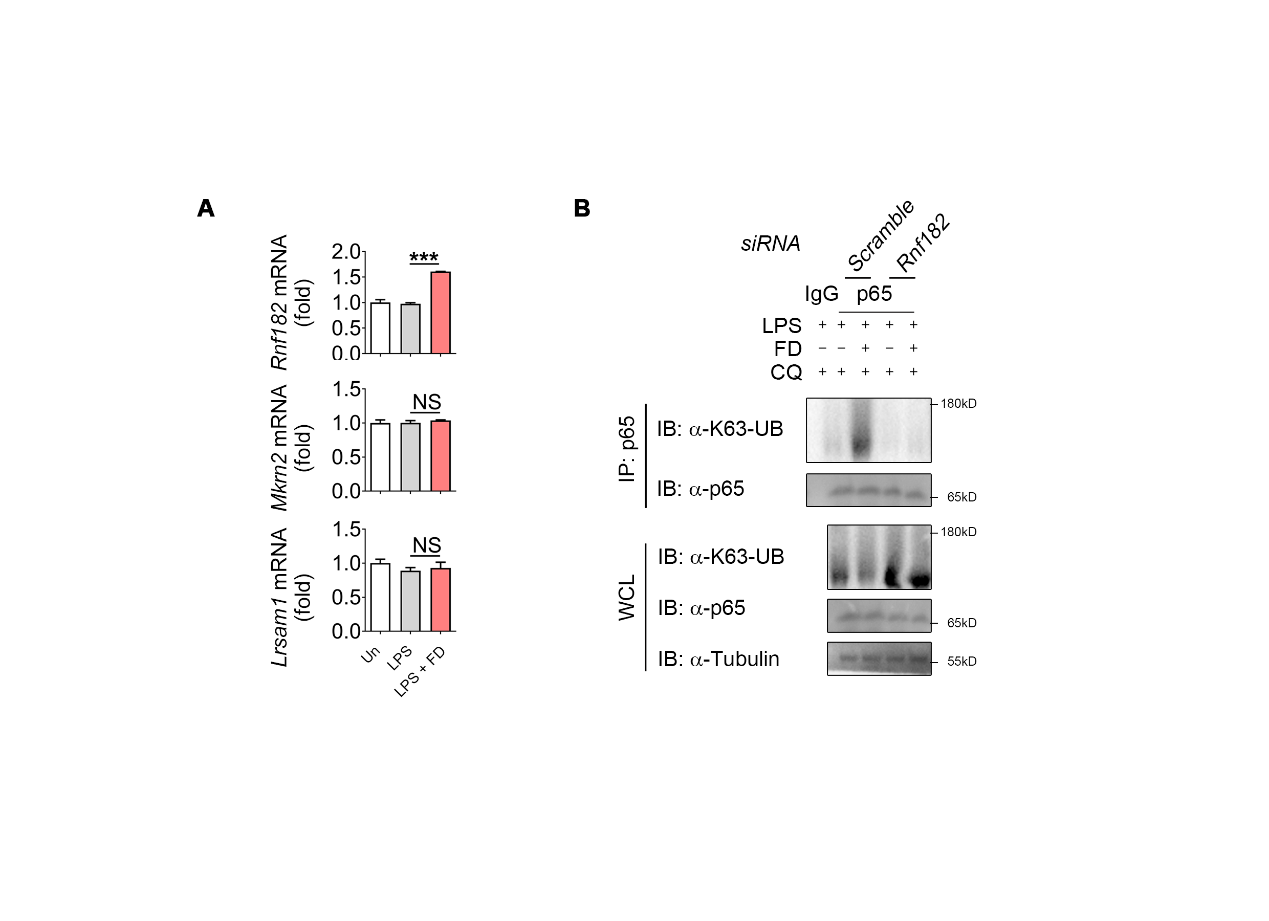


**Figure S5.** FD increases the interaction p65 and RNF182, and increases the polyubiquitination of p65. (A) LPS-induced mice NP cells were treated with diverse concentrations of FD for 12 h. Quantitative PCR analysis of *Rnf18, Mkrn2, Lrsam1* in LPS-induced rat NP cells treated with or without FD. (B) NP cells underwent Lrsam1 siRNA silencing for 24 h. And the cells were stimulated with CQ (50 μM) for 6 h and exposed to LPS (100 ng/ml) and FD (4 μM) for 8 h. The cell lysates were subjected to immunoprecipitation with an anti-p65 antibody or control IgG and immunoblotted with the indicated antibodies. NS, not significant; ***p<0.001.


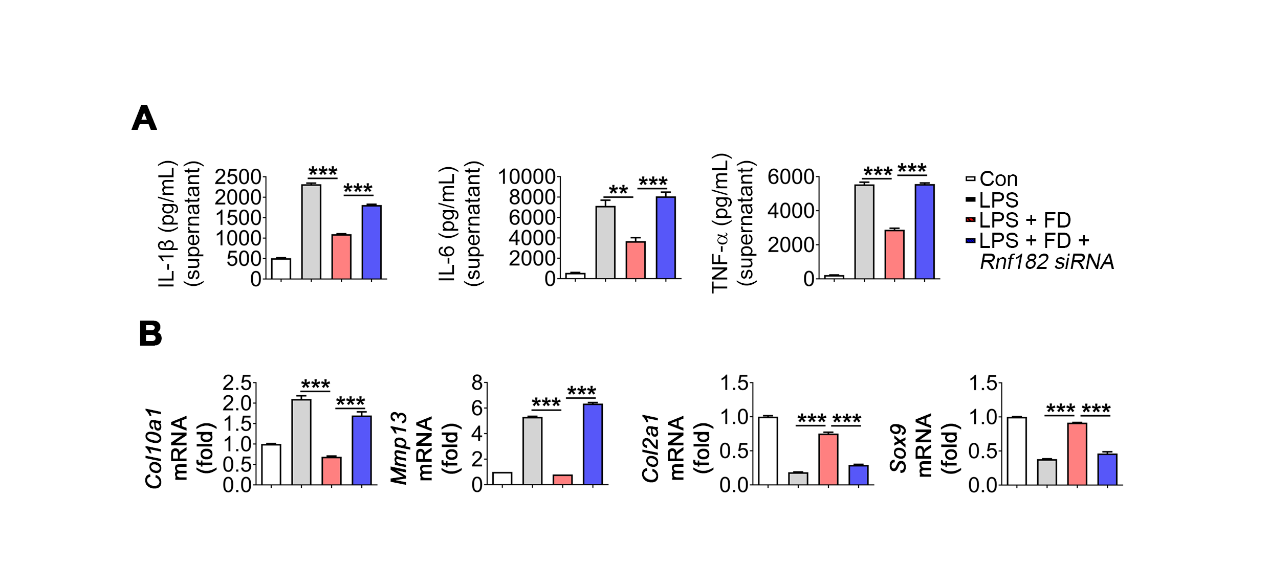


**Figure S6.** FD reduces inflammation and NP destruction by recruiting RNF182. (A) ELISA analysis of IL-1β, IL-6 and TNF-α levels in the supernatant of LPS-induced NP cells treated with vehicle, FD or FD plus *Lrsam1 siRNA*. (B) NP cells were co-cultured with CM, vehicle-treated M1 macrophage CM, FD-treated NP cells CM or FD plus *Lrsam1 siRNA*-treated NP cells CM for 24 h. Quantitative PCR analysis of Col10a1, MMP13, Col2a1 and Sox9 in NP cells. ***p<0.001.
